# Community Evolution in International Migration Top1 Networks

**DOI:** 10.1371/journal.pone.0148615

**Published:** 2016-02-09

**Authors:** Mihaela Peres, Helian Xu, Gang Wu

**Affiliations:** 1 School of Economics and Trade, Hunan University, Changsha, China; 2 School of International Business, Southwest University of Finance and Economics, Chengdu, China; Cinvestav-Merida, MEXICO

## Abstract

Focusing on each country’s topmost destination/origin migration relation with other countries, this study builds top1 destination networks and top1 origin networks in order to understand their skeletal construction and community dynamics. Each top1 network covers approximately 50% of the complete migrant network stock for each decade between 1960 and 2000. We investigate the community structure by implementing the Girvan-Newman algorithm and compare the number of components and communities to illustrate their differences. We find that (i) both top1 networks (origin and destination) exhibited communities with a clear structure and a surprising evolution, although 80% edges persist between each decade; (ii) top1 destination networks focused on developed countries exhibiting shorter paths and preferring more advance countries, while top1 origin networks focused both on developed as well as more substantial developing nations that presented a longer path and more stable groups; (iii) only few countries have a decisive influence on community evolution of both top1 networks. USA took the leading position as a destination country in top1 destination networks, while China and India were the main Asian emigration countries in top1 origin networks; European countries and the Russian Federation played an important role in both.

## Introduction

Cross-border migration is regarded as a fundamental characteristic of human life and has turned into a significant force all over the world with important economic, social, and political implications [1]. Moreover, with a constant growth throughout the last five decades of the twentieth century from 93 to 167 million [2], global migration reached 232 million people living outside their country of origin in 2013, and this figure is forecasted to double by 2050 [3]. Even more essential is the fact that the increasing complexity of migratory patterns—global mobility influencing from individual through families, industries, countries, along with the possibility to redesign the world where we live—have led migration to become a priority for international communities [4].

**Fig 1:**
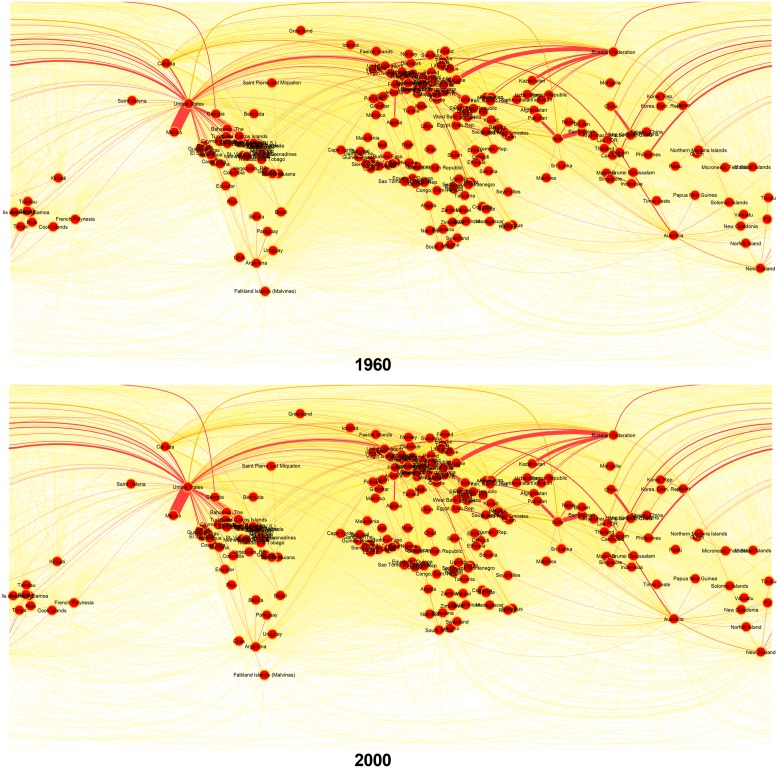
Complete International Migration Network in the years 1960 and 2000. The plots in the figure shows the direct weighted version of the CIMN that highlights the top-ranked destination of each country. The colours of the links represent the proportion of migrant stock in the maximum migrant stock after country of destination [*wij* / max *i*(*wij*)] from light yellow (low-proportion links) to red (high-proportion links). The thickness of the links is proportional to the normalized migrants stocks [*wij* / max(*wij*)].

Despite the fact that the size and complexity of the migration phenomenon has been growing, most studies have remained confined to the level of the country or on the level of country-to-country flows [5]. Even if in the vast majority of these scientific studies the migration has been regarded as a bilateral phenomenon, the database and the methodology used only offer a small piece of the whole picture. To overcome these limitations, we combined the graph-theoretical methods as a basic concept along with a global migration data set [2]; this allows us to obtain an overview of migration by illustrating this complex system with a mature and well-understood approach. We use R software [6] to visualize the complete international migration network in the years 1960 and 2000 (see Fig 1). Analysing the properties of this network is crucial for comprehending the migration patterns and for offering a review of migration; it can be used to show how changes occurring at a node can influence the behaviour and importance of other apparently unrelated nodes.

The network perspective approach has gained increased attention from a growing number of researchers interested in examining the structural and dynamical properties involving networks in a wide variety of disciplines [7, 8], including the development of social network analysis in sociology [9], economics [10], human-mobility in general [11–13], and many more. Moreover, a key question in network science concerns the topological measures utilized to define the properties of the network connecting the agents, and in what way these properties influence the behaviour of the agents as well as the evolution of the system analysed [10].

However, in terms of migration, only a few researchers have tried to recast the trend by employing a network viewpoint [14–17]. Making use of a global origin-destination migration matrix [2], some scholars characterize directed (i.e., binary and weighted) and undirected architectures of the network to better understand the network’s structure: either it has remained relatively stable through the years with very skewed distributions for weighted links and node statistics [16], or the structure has undergone a steady increase in network transitivity, or the structure has experienced a decrease of the average path length with an upward shift in degree distribution [17]. Both studies reinforce the idea of “small-world” behaviour of the migration network. Considering the algorithm developed by [18], known as the Louvain method (i.e., aiming to maximise modularity in a network), some scholars focus their work on exploring the network topology and then uncover the communities formed by OECD countries in the migration network [15]. In the same manner, researchers are interested in the relationship as well as the effect of human mobility on international trade [19, 20] and also on country income and labour productivity [21]. Even if these studies provide an important contribution to the literature concerning the deepening community structure and evolution of the migration network, they have not proposed an exclusive radiography of the communities seen from the perspective of both country of origin and country of destination.

In addition to these shortcomings, not all migration relations are equally important for a country; therefore, in this paper we propose the extraction of international migration network based on each country’s topmost migration stock [22] with other countries (that is, country *i* is linked to country *j* only if *j* is *i’s* most important migration country; otherwise, there is no link between *i* and *j*). Specifically, we built the top1 destination network Top1D (that is, country *i* is linked to country *j* only if *j* is *i’*s top1 destination country for emigrants) and the top1 origin network Top1O (that is, country *j* is linked to country *i* only if *i* is *j’*s top1 origin country for immigrants). These two networks capture the most important relationships of the complete international migration network and cover approximately 50% from the complete migrant network stock. This is the first study that builds and analyses international migration networks based on top migration relationships.

Further, we determine the structure and evolution of the communities. For each decade between 1960 and 2000, the two top1 networks present nearly 80% stability in terms of top1 relationships and exhibit communities with a clear structure but with a surprising evolution. The remaining (20%) are strong enough to promote changes in the communities; they show an opposite trend concerning the number of clusters. In other words, featuring a decrease in the number of communities between 1960 to 2000, Top1D focused on developed countries and exhibited shorter paths and preferred more advanced countries. Over the same time period, with an increasing number of communities, Top1O focus more on both developed as well as more substantial developing nations; this presents a longer path and more stable groups.

Furthermore, by exploring the composition of the communities, we notice that countries with traditions in receiving immigrants (e.g., USA) experienced a change in terms of the composition and amount of migrants compared with a more stable situation presented in Australia and New Zealand. World War II was followed by independence for most former colonies of European and Japanese empires [23]. The dominating key source of migrants, Europe, was replaced by Africa, Asia, Latin America, and the Caribbean. Instead of their empires, societies like Britain and France created links with those former colonies, which fostered migration among them and changed their position into immigrant-receiving countries. Japan, with a declining birth rate and aging symptoms, was in a position to import immigrants from poorer countries of Asia and South America in order to meet the workforce needs [1]. Taking into consideration the effects generated, comprehending the evolution of international migration communities can reveal more about the migration patterns.

## Materials and Methods

### Data and Complete International Migration Network (CIMN)

The source for data employed in this paper is United Nations Population Division's Global Migration Database [2], which consists of an origin-destination square matrix tracking bilateral migration between 231 countries for each decade between 1960 and 2000 (e.g., 1975 through 1984 is assigned to 1980). Just one standard list of countries is chosen for the entire time span of this database, regarding both origin and destination countries; this allowed comparison of the migration figures over time. For example, the 15 new sovereign states created after the break-up of the Soviet Union were treated as separate countries at every decade between 1960 and 2000 (that is, the internal migrants during the years of the Soviet Union in this database were considered as international migrants) [2]. This unique dataset comprises 3500 individual census and population records and provides information on international migrant bilateral stocks [2]. Preferentially, country of birth is used to define country of origin, and migration data are firstly provided by the destination country [2].

Bilateral data included in this dataset refer to immigrant stock instead of flows to facilitate interpretation [24]. Furthermore, data for bilateral flows are only available for OECD countries, which obviously limit the overall coverage considerably [25]. Therefore, seeking to provide an overview of the migration phenomenon covering a larger number of countries, we chose to use the migrant stock [14, 16, 17, 19, 20]. An important note is that this database does not include two important aspects of migration, that is, illegal and within border (internal) migration.

Nearly all systems analysed today are constructed from many elements; each have an independent role but make contributions to the whole. By considering only the degree of a node, we ignore the proven fact that even the nodes with small degrees can play an important role in connecting different regions of the network by servings as bridges. Another neglected aspect is related with losing the possibility of emphasizing the relationships between countries and groups of countries.

Starting from the idea that relationships as well as connections are some of the most significant components that characterize the shape and the conduct of the physical and social world as we comprehend it, we begin our analysis with the fact that international migration can be treated as a network of nodes (i.e., countries) that are connected via links that represent the migrants stock. Inside the origin-destination matrix, states like Channel Islands, Isle of Man, Kosovo, Montenegro and Serbia do not share any relationships with other nodes so we reduce the database to 226 countries [14, 16, 17, 19, 20].

Given these issues, we define the complete international migration network (CIMN) as a weighted, directed network: $M_{{}_{}}^{t}={{\{\text{w}}_{{}_{\text{ij}}}^{t}\}}_{\text{N}\times \text{N}}$ where *M*^*t*^ represents the matrix, time is year *t =* [1960; 1970; 1980; 1990; 2000], countries are *N* = 226, and ${\text{w}}_{{}_{\text{ij}}}^{t}$ is the stock of migrants born in country *i* living in destination country *j* at the time *t*.

Accordingly, we define the binary projection of the international migration network as a directed network $A_{{}_{}}^{t}={{\{\text{a}}_{{}_{\text{ij}}}^{t}\}}_{\text{ N}\times \text{N}}$, where ${\text{a}}_{{}_{\text{ij}}}^{t}=\left\{ \begin{array}{l} {\text{1, w}}_{\text{ij}}^{\text{t}}>0;\\ 0,{\text{w}}_{\text{ij}}^{\text{t}}=0; \end{array}\right.$, and ${\text{a}}_{{}_{\text{ij}}}^{t}$ expresses the presence of migrants born in country *i* living in destination country *j* at time *t*.

International migration provides an impressive network that encompasses countries connected by several links of cross-border movements. Compared with the initial facts (e.g., the total number of immigrants living in each country), the analysis of entire network characteristics gives an integrated knowledge of human migration and shows how changes in behaviour of a node can influence other apparently unrelated nodes.

### Top1 networks

CIMN is represented by a huge cluster with a large number of links established between network nodes. For each country, we rank its migration relationships with other countries by the number of migrant stock because some migration relationships are more important than others, especially the ones ranked first, which are called top1 [22].

The top1 network comprise each country’s topmost migration relationships (the strongest link) with other countries, the top2 network comprise each country’s top two migration relationships (the strongest and second strongest link) with other countries, and so on. The topmost important edges included in the top1 network covered approximately 50% of the complete migrant network stock. Meanwhile, the percentage of the top2 and top3 are around 61% and 69%, respectively. These percentages are generally stable over time. The impressive percentage of the top1 network captured our attention. For example, consider the data for international migration in 1960. When only the strongest tie for each country is kept, the top1 network will include only 226 links (from a total of 16485 links) but will cover around 46.5 million migrant stock (that is, around 50% of the total 93 million).

Therefore, starting from the weighted directed matrix *M*^*t*^ and considering each country’s topmost migrant stock born in country *i* and living in destination country *j*, we build two top1 networks. The first is the international migration top1 destination network (Top1D) and is defined as follows:

$${\text{Top1D}}_{{}_{}}^{t}={{\{\text{dw}}_{{}_{\text{ij}}}^{t}\}}_{\text{N}\times \text{N}},\;dw_{{}_{\text{ij}}}^{t}=\left\{ \begin{array}{l} {\text{w}}_{ij}^{t}{\text{, w}}_{\text{ij}}^{\text{t}}=\underset{j}{\text{max}}({\text{w}}_{\text{ij}}^{\text{t}})\\ 0,otherwise; \end{array}\right.,$$ (1)

where $dw_{{}_{\text{ij}}}^{t}$ is the max migrant stock born in *i* living in destination *j* at time *t* after country of origin *i*.

Accordingly, we define the binary projection of Top1D as follows:

$${\text{Top1AD}}_{{}_{}}^{t}={{\{\text{da}}_{{}_{\text{ij}}}^{t}\}}_{\text{N}\times \text{N}}$$ (2)

We define the second by extracting the international migration top1 origin network (Top1O):

$${\text{Top1O}}_{{}_{}}^{t}={{\{\text{ow}}_{{}_{\text{ij}}}^{t}\}}_{\text{N}\times \text{N}},\;ow_{{}_{\text{ij}}}^{t}=\left\{ \begin{array}{l} {\text{w}}_{ij}^{t}{\text{, w}}_{\text{ij}}^{\text{t}}=\underset{i}{\text{max}}({\text{w}}_{\text{ij}}^{\text{t}})\\ 0,otherwise; \end{array}\right.,$$ (3)

where $ow_{{}_{\text{ij}}}^{t}$ represents the max migrant stock born in *i* living in destination *j* at time *t* after country of destination *j*.

The binary projection of Top1O is defined as follows:

$${\text{Top1AO}}_{{}_{}}^{t}={{\{\text{oa}}_{{}_{\text{ij}}}^{t}\}}_{\text{N}\times \text{N}}$$ (4)

One crucial attribute of the top-ranked networks refers to the out- and in-degree. Specifically, in top destination networks, all nodes have an out-degree of the selected standard but with varying in-degrees across countries. As an example, in the top1 destination network, all the countries have out-degrees equal to 1, while the in-degrees vary across the nodes and can be greater than 1. This means that a country can have only one biggest origin source but can be the destination for many other countries. Of course, if the country is not selected as a destination, the number of in-degrees will equal 0. On the other hand, in top origin networks, all the countries have an in-degree of the selected standard but their in-degree varies across the countries.

**Fig 2:**
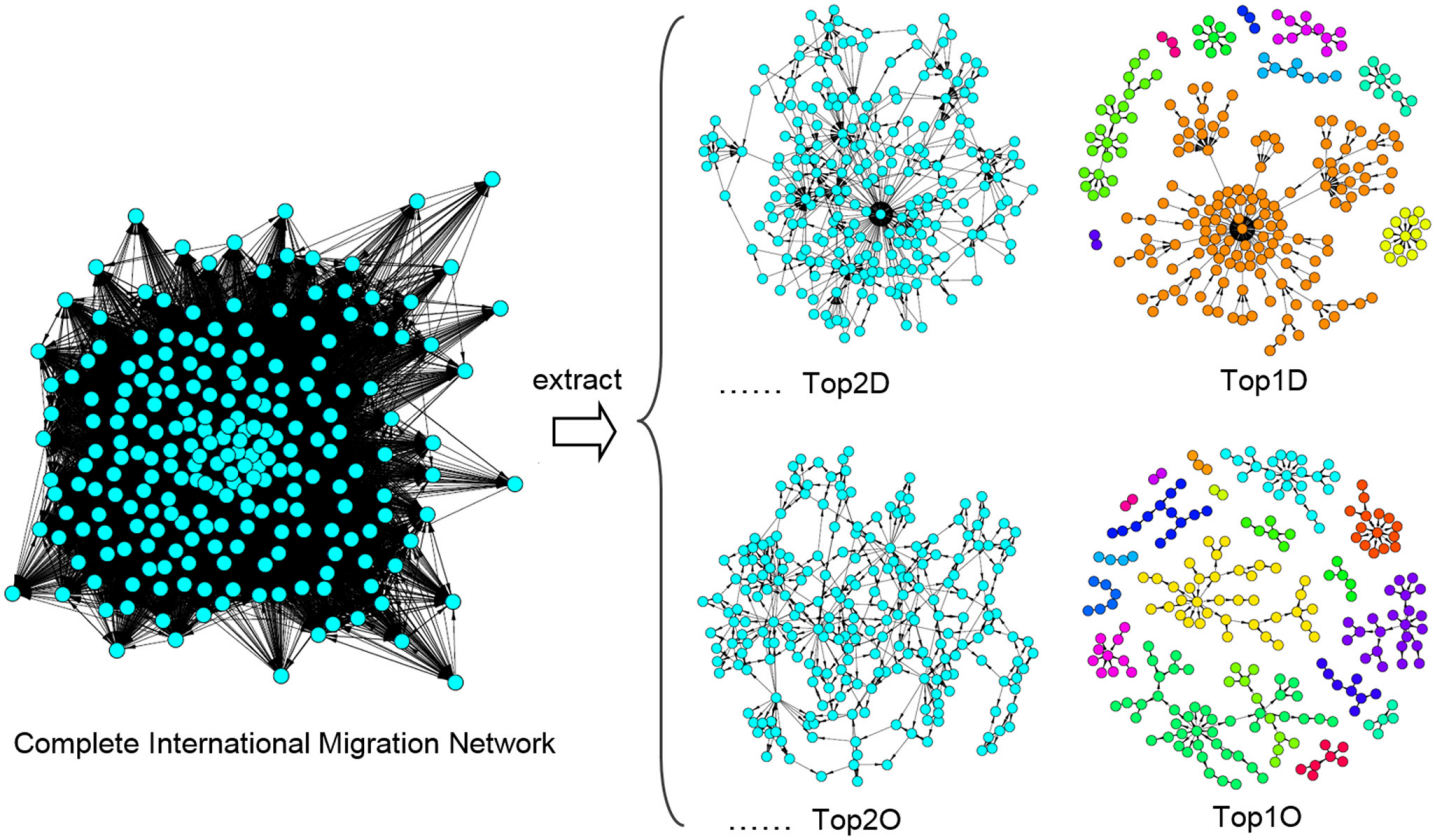
Extracting international migration top networks from the CIMN in 2000.

Fig 2 shows these networks using international migration data for the year 2000. To construct this figure, we began with the complete international migration network. Next, we kept each country’s strongest migration link and extracted Top1D (the strongest outgoing link) and Top1O (the strongest incoming link). In the same way, Top2D and Top 2O networks can be extracted by keeping each country’s top two important migration ties (the strongest and second strongest link), etc.

### Network measures

By computing the basic information for all the nodes from the network, the topology can be characterized through selected metrics [14, 26, 27]. Probably the most widespread and accepted of these metrics include density (i.e., the ratio of the number of edges and the number of possible edges), diameter (i.e., the maximum shortest path length in a network), average path length (*APL*, i.e., the average length of all the shortest paths from or to the vertices in the network, considering directed paths in directed graphs), and degree (*ND*, i.e., the number of its adjacent edges). In directed graphs, the degree of a node is defined by the sum of its in-degree (*ND*_*in*_) and its out-degree (*ND*_*out*_), *ND*_*i*_ = *ND*_*in*,*i*_ + *ND*_*out*,*i*_, where the in-degree *ND*_*in*,*i*_ of the node *i* is defined as the number of edges going to *i*, and its out-degree *ND*_*out*,*i*_ is defined as the number of edges exiting from *i*. In terms of the adjacency matrix, we can write the following:

NDin,i=∑jAji, NDout,i=∑jAij (5)

For the original non-symmetrical matrices, the strength or weighted vertex degree is calculated as summing the edge weights of the adjacent edges for each vertex (i.e., country). Mode is defined as “out” for out-degree and “in” for in-degree.

Considering these metrics and their evolution will reveal how the composition of migration changed due to world events and progressively more selective immigration regulations in developed countries. As an overview, the migration process is influenced at every decade by the globalization phenomenon.

To make the comparison between both top1 networks and to emphasize their importance in the CIMN, we built a global level index at every decade:

$${\text{pTop1D}}_{{}_{}}^{t}=\frac{\sum_{\text{i}}\sum_{j}{\text{dw}}_{\text{ij}}^{\text{t}}}{\sum_{\text{i}}\sum_{\text{j}}{\text{w}}_{\text{ij}}^{\text{t}}}$$ (6)

$${\text{pTop1O}}_{{}_{}}^{t}=\frac{\sum_{\text{i}}\sum_{j}{\text{ow}}_{\text{ij}}^{\text{t}}}{\sum_{\text{i}}\sum_{\text{j}}{\text{w}}_{\text{ij}}^{\text{t}}}$$ (7)

where pTop1D^*t*^ and pTop1O^*t*^ represents the proportion of each top1 network in the CIMN at time *t*.

Furthermore, in order to check the proportion of persistent edges in top1 networks, we built the following index:

$${\text{pSETop1D}}_{{}_{}}^{t}=\frac{\sum_{\text{i}}\sum_{j}\text{(1}|{\text{da}}_{\text{ij}}^{\text{t}}=d{\text{a}}_{\text{ij}}^{\text{t-1}}=1)}{\sum_{\text{i}}\sum_{\text{j}}{\text{da}}_{\text{ij}}^{\text{t}}}$$ (8)

$${\text{pSETop1O}}_{{}_{}}^{t}=\frac{\sum_{\text{i}}\sum_{j}\text{(1}|{\text{oa}}_{\text{ij}}^{\text{t}}=o{\text{a}}_{\text{ij}}^{\text{t-1}}=1)}{\sum_{\text{i}}\sum_{\text{j}}{\text{oa}}_{\text{ij}}^{\text{t}}}$$ (9)

where pSETop1D^*t*^ and pSETop1O^*t*^ emphasize the stability of edges in each top1 network and represents the proportion of persistent edges from previous phase in total edges.

### Communities

Despite the significant role of human migration as a major contributor to globalization, the understanding of community structure and evolution inside the top1 networks remain poorly understood.

In most real-world networks, especially in social networks, the node has a tendency to create properly knitted groups with a relatively high density of ties [28, 29]. The literature provides several definitions and methods to detect communities. As a result, most algorithms can be distinguished into categories of divisive [30], agglomerative [31], and optimization-based [32]. Linkage-based approaches exploit the topological information of a network to identify dense sub-graphs.

Among the available detection algorithms for a network with directed-weighted edges, community detection based on the edge-betweenness algorithm is the most suitable [30]. Thus, using the Girvan-Newman algorithm [30, 33], we detect the communities in both top1 networks. The betweenness centrality of edges can be calculated analogously to the node betweenness of the number of shortest paths among all possible node pairs that pass through a given edge. The edges with a maximum score are assumed to be more important for a graph to remain interconnected. Granovetter called these edges “weak ties” that interconnect clusters of nodes [34]. The algorithm computes the edge betweenness of the graph by removing the edge with the highest edge betweenness score, then it recalculates the edge betweenness of the edges and again removes the one with the highest score, etc.

## Results and Discussion

### CIMN and Top1 Networks: Descriptive statistics

Due to the fact that migration occurs inside the network, examining its characteristics is essential for comprehending migration patterns. The changes in dynamics, through the appearance and disappearance of some links or perhaps through short-cuts capable to avoid a longer path, affect the architectures of the networks between each two consecutive decades. The top1 networks extract the most important links from the overall international migration network. Accordingly, the migrant stock in the top1 destination network and the top1 origin network make up around 50% of the total global migrant stock. The percentages of Top1D vary from 53.6% in 1960 to 48% in 2000 compared with Top1O, which varies from 54.1% in 1960 to 35.4% in 2000; this once again shows the impact of globalization on the phenomenon of migration.

Considering its distinct relevance, we have taken the step of investigating the structure and evolution of the complete international migration network and both top1 networks.

Table 1 presents basic descriptive statistics about the complete international migration network in ten-year intervals spanning the years 1960–2000. Furthermore, with a constant number of nodes (equal with 226) along the five networks, the CIMN features both extensive and intensive growth. First, the number of links between countries grew 45% from 16485 in 1960 to 23718 in 2000. As an average, at every decade, around 1446 new links were established between pairs of countries. This has resulted not only in an increase in density from 0.324 to 0.466 but also an increase in the mean node degree from 145.9 to 209.9, while the average path length decreased from 1.749 to 1.535. Further, regarding the maximal in-degree and out-degree, the situation shows the same pattern reaching the highest level in 2000 when a node received migrants from almost all the countries of the network (i.e., 223) and sent 216. Second, the number of migrant stock increased remarkably. The mean node strength (expressed in thousand) increased from 5.646 to 7.044 migrants, recording a maximal value of 34814.064 in destination country for the year 2000 and 13244.244 in the origin country for the year 1990.

**Table 1:** Descriptive statistics regarding the complete international migration network.

Year	1960	1970	1980	1990	2000
No. Nodes	226	226	226	226	226
No. Edges	16485	18110	19319	21731	23718
Density	0.324	0.356	0.38	0.427	0.466
No. Components	1	1	1	1	1
Diameter	4	4	4	4	3
APL	1.749	1.697	1.669	1.607	1.535
CC	0.673	0.693	0.713	0.737	0.755
Mean ND	145.9	160.3	171	192.3	209.9
Max. ND_*in*_	218	217	220	219	223
Sd. ND_*in*_	49	49.7	50.5	51.1	53.1
Max. ND_*out*_	205	206	209	208	216
Sd. ND_*out*_	43.4	45.6	46.5	48.5	48.3
Mean NS	5.646	5.842	6.221	6.528	7.044
Max. NS_*in*_	10825.585	11973.797	16364.414	23251.023	34814.064
Sd. NS_*in*_	1243.763	1376.715	1629.894	2025.225	2705.622
Max. NS_*out*_	9081.881	10565.229	11682.097	13244.244	10375.787
Sd. NS_*out*_	1254.811	1291.285	1266.128	1380.501	1432.812
pSE	---	0.851	0.866	0.830	0.843

Notes: APL: Average path length; CC: Clustering coefficient; ND: Node degree; ND_*in*_: Node in-degree; ND_*out*_: Node out-degree; NS: Node strength; NS_*in*_: Node in-strength; NS_*out*_: Node out-strength. Units for NS, NS_*in*_, NS_*out*_ are in thousands of migrant stock. pSE: Proportion of persistent edges from previous phase in total edges in CIMN $\left(\sum_{i}\sum_{j}(1|a_{ij}^{t}=a_{ij}^{t-1}=1)/\sum_{i}\sum_{j}a_{ij}^{t}\right)$.

The diameter of a growing random network can be more different than, for example, the one of a Poisson random network. This growing network has large-degree nodes that emerge and may work as hubs to reduce the overall distance between countries [35]. With a small, almost constant diameter displaying a decreasing average path length and an increasing clustering coefficient [36], we conclude that the CIMN demonstrates a “small-world” behaviour [37, 38].

Top1 migrant relations are by definition the most important migrant ties for countries. In the Top1D network, by definition each country can have only 1 out-degree, but they can have different in-degrees. In this way, the in-degree determines a country’s position in the network. Similarly, in a Top1O network, each country can have only 1 in-degree, but they can have several out-degrees, which will determine how central a country is. As a result, the number of nodes will equal the number of edges. The only exception to this rule is found in the Top1O network, where in the first four decades some countries are not considered as destinations. For example, in the 1960’s and 1970’s, those countries were Norfolk Island and Taiwan (reducing the number of edges at 224) and in the 1980’s and 1990’s, those countries were Taiwan and Belize (reducing to 225).

Both top1 networks share the same number of countries (i.e., 226) showing a small density (i.e., 0.0004) caused by the reduced number of ties between countries and a mean node degree maintained at a constant value of 2.

Table 2 presents basic descriptive statistics about the top1 destination networks for each decade between 1960 and 2000, where the five networks are represented by disconnected graphs with a number of components that vary over time from 14 to 10 and have an infinite diameter [39].

**Table 2:** Descriptive statistics about the top1 destination network.

Year	1960	1970	1980	1990	2000
No. Nodes	226	226	226	226	226
No. Edges	226	226	226	226	226
Density	0.004	0.004	0.004	0.004	0.004
No. Components	14	14	9	9	10
Diameter	Inf	Inf	Inf	Inf	Inf
APL	2.162	2.04	1.954	1.906	1.95
Mean ND	2	2	2	2	2
Max. ND_in_	37	32	58	58	60
Sd. ND_in_	3	2.8	4.3	4.3	4.4
Max. ND_out_	1	1	1	1	1
Sd. ND_out_	0	0	0	0	0
Mean NS	220.778	241.295	273.915	310.645	354.860
Max. NS_in_	9274.493	8845.067	10921.398	16584.967	25270.152
Sd. NS_in_	1000.801	1046.676	1230.917	1507.996	1967.236
Max. NS_out_	8662.538	8141.307	4803.152	5211.922	9367.910
Sd. NS_out_	807.970	768.144	678.792	716.162	832.822
pTop1D	0.536	0.515	0.515	0.495	0.480
pSETop1D	---	0.642	0.615	0.761	0.761

Notes: APL: Average path length; CC: Clustering coefficient; ND: Node degree; ND_*in*_: Node in-degree; ND_*out*_: Node out-degree; NS: Node strength; NS_*in*_: Node in-strength; NS_*out*_: Node out-strength. Units for NS, NS_*in*_, NS_*out*_ are in thousands of migrant stock. pTop1D: Proportion of Top1D network as defined in Eq (6); pSETop1D: Proportion of persistent edges in Top1D network as defined in Eq (8).

Moreover, with a maximal node in-degree growing throughout the five networks from 37 in 1960 to 60 in 2000, the first migrant receiving country in the world is USA, which presents an increasing immigrant stock from 9274.493 in 1960 to 25270.152 in 2000. The maximal node out-degree, presented in Table 3, reveal a different situation concerning the most important migrant-sending countries. The first three networks indicate that the United Kingdom is the biggest source for emigrant stock, growing from 8685.810 in 1960 to 10416.947 in 1980. British emigrants were the largest migrant stock for 16 nodes in 1960 and for 18 nodes in 1970 and 1980. The last two networks indicate the Russian Federation as a the most important migrant-sending country to 15 nodes, with a decreasing emigrant stock from 11416.527 in 1990 to 9367.910 in 2000. Furthermore, Top1O networks, similar to Top1D networks, are represented by disconnected graphs with infinite diameter but with an increasing number of components from 13 in 1960 to 19 in 2000.

**Table 3:** Descriptive statistics about the top1 origin network.

Year	1960	1970	1980	1990	2000
No. Nodes	226	226	226	226	226
No. Edges	224	224	225	225	226
Density	0.004	0.004	0.004	0.004	0.004
No. Components	13	17	16	20	19
Diameter	Inf	Inf	Inf	Inf	Inf
APL	2.713	2.152	2.333	2.32	2.453
Mean ND	2	2	2	2	2
Max. ND_in_	1	1	1	1	1
Sd. ND_in_	0.1	0.1	0.1	0.1	0
Max. ND_out_	16	18	18	15	15
Sd. ND_out_	2.2	2.2	2.3	2	2
Mean NS	224.873	230.841	225.600	247.021	261.962
Max. NS_in_	8662.538	8141.307	4803.152	5211.922	9367.910
Sd. NS_in_	816.585	774.675	651.571	703.098	806.511
Max. NS_out_	8685.810	9257.766	10416.947	11416.527	9367.910
Sd. NS_out_	1051.598	1047.357	990.099	1064.278	1047.385
pTop1O	0.541	0.489	0.422	0.392	0.354
pSETop1O	---	0.826	0.844	0.853	0.836

Notes: APL: Average path length; CC: Clustering coefficient; ND: Node degree; ND_*in*_: Node in-degree; ND_*out*_: Node out-degree; NS: Node strength; NS_*in*_: Node in-strength; NS_*out*_: Node out-strength. Units for NS, NS_*in*_, NS_*out*_ are in thousands of migrant stock. pTop1O: Proportion of Top1O networks, as defined in Eq (7); pSETop1O: Proportion of persistent edges in Top1O network as defined in Eq (9).

The most important migrant ties for countries exhibits a maximal node strength (that is, maximal large share of stock) of 8662.538 thousands in 1960 and 8141.307 in 1970 reflecting the partition of India and her immigrants coming from Pakistan. The largest immigrant stock in the 1980 (that is, 4803.152 thousand) and 1990 (that is, 5211.922 thousand) networks were registered in Ukraine with the Russian Federation as the origin country. All networks under study exhibited Mexico as the leading origin country and USA as the leading destination country with a total of 9367.910 thousand migrants.

The proportion of persistent edges indicate a high stability over time for all the three networks (i.e., CIMN, Top1D, and Top1O). CIMN and Top1O exhibit high stability with similar results in both networks. The average of the stability of edges in CIMN was around 0.85, evolving from 0.851 in 1970 to 0.843 in 2000, while, with an evolution from 0.826 in 1970 to 0.836 in 2000, Top1O recorded an average of 0.84. Meanwhile, Top1D displayed a lower stability (that is, the average of 0.70), evolving from 0.642 in 1970 to 0.761 in 2000.

### Communities vs. Components

In addition to the analysis of migrant stock dynamics, information about the connections of each node can be used to identify the community structure, i.e., the existence of clusters. For the purpose of determining the number of components, we ignore a key feature of our networks, namely, the edges’ weight. Therefore, in order to overcome this limitation, we add the weight and then apply the community-detection Newman-Girvan modularity algorithm to explore the communities’ structure. Specifically, we use the i-graph package in R [6, 40] to detect the communities for each decade between 1960 and 2000 and to investigate the underlying behaviour of the network.

Afterwards, we analyse the dynamics of these communities to understand their evolution over time as well as the possible gradual disappearance of the legacy involving old communities within the following decades.

**Fig 3:**
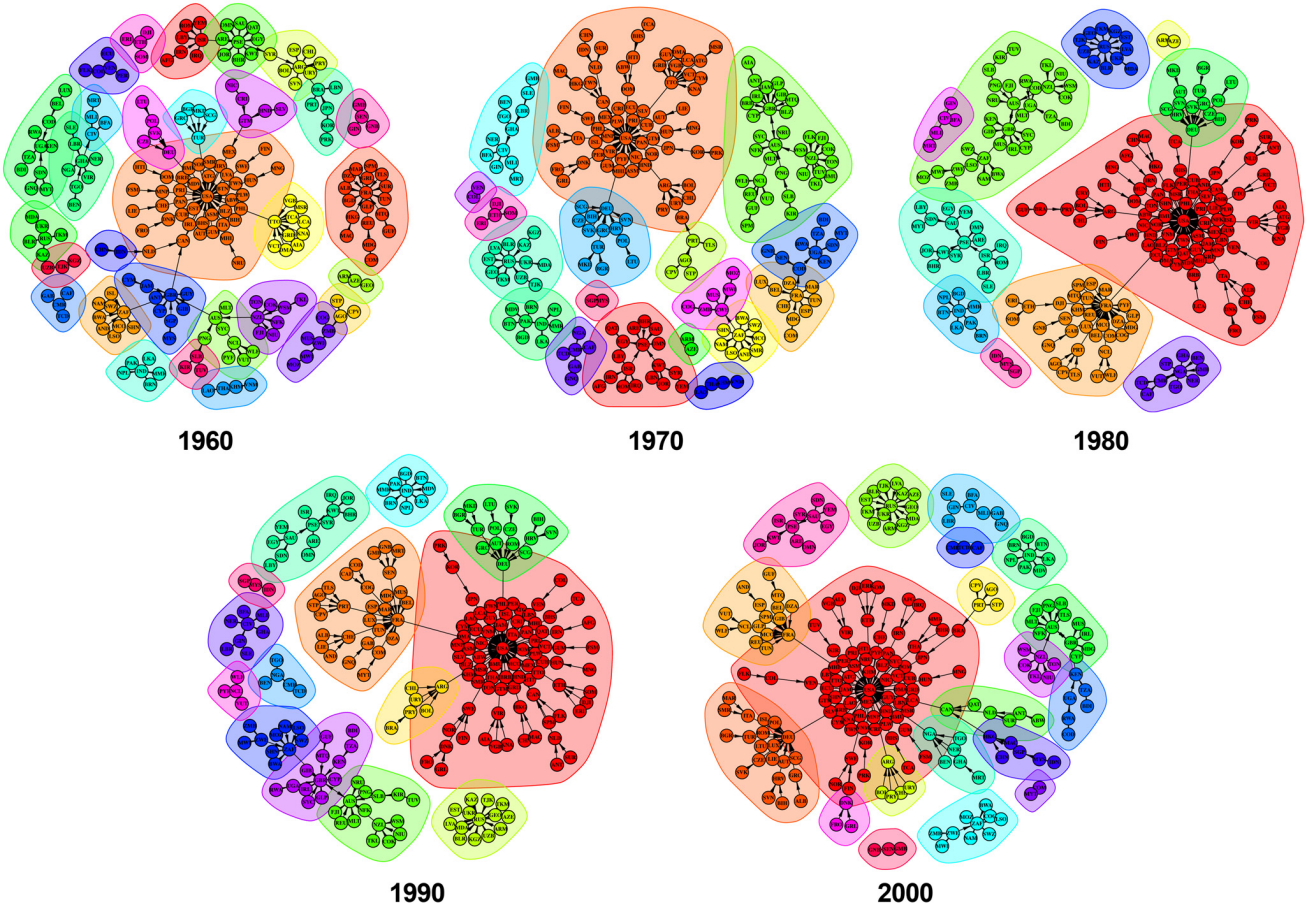
Communities detection in top1 destination network for each decade between 1960 and 2000. The nodes having the same colour are members of the same component, while the same background shows that they belong to the same community.

Fig 3 shows in an accessible manner the evolution of the communities inside Top1D networks. Once the weight characteristic was added to the edges, the structure of the network changed, showing a decreasing evolution in the number of communities throughout time.

**Fig 4:**
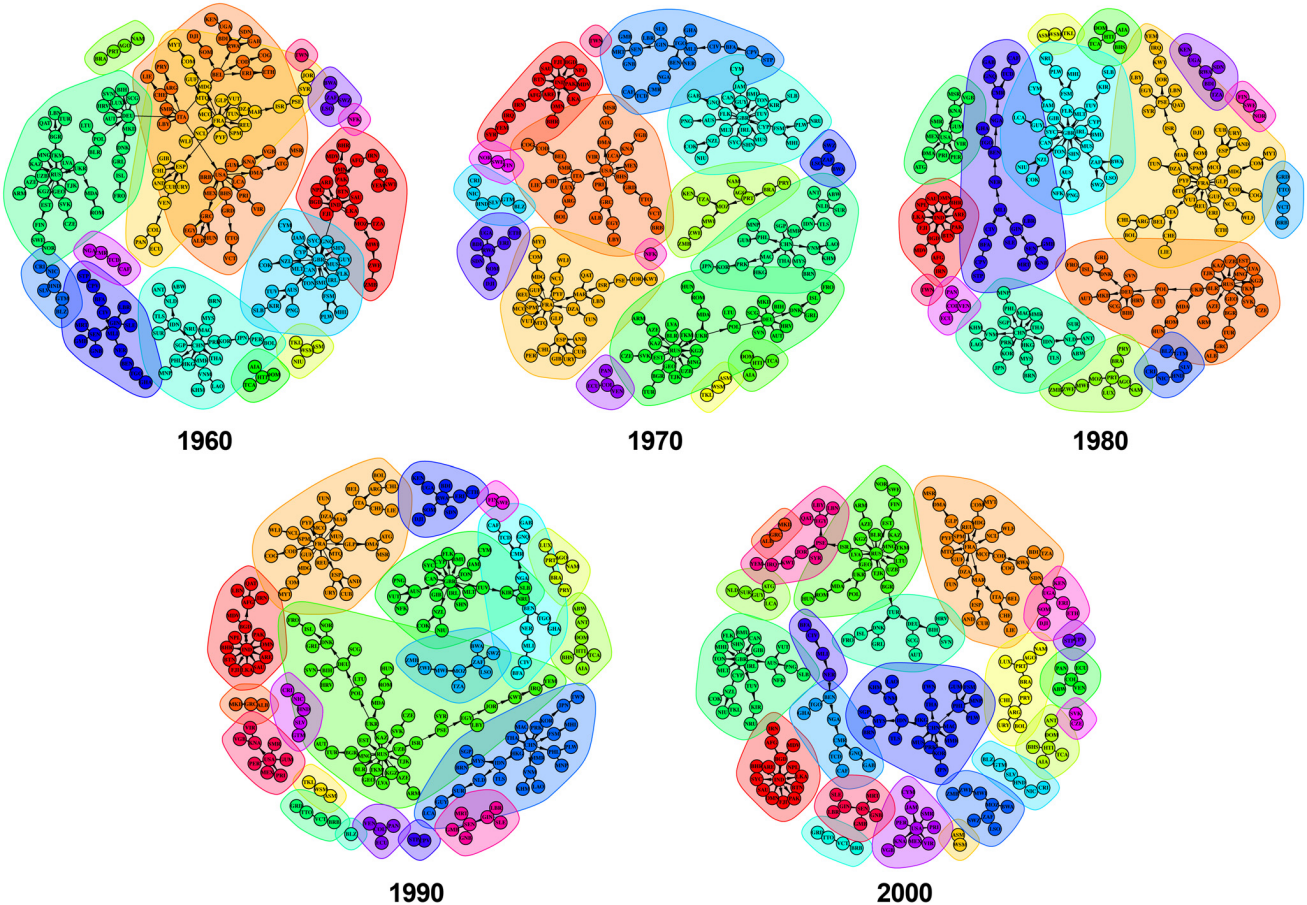
Communities detection in top1 origin network for each decade between 1960 and 2000. The nodes having the same colour are members of the same component, while the same background shows that they belong to the same community.

In contrast, Fig 4 presents the opposite trend of Top1O networks, with an increasing number of communities during the time span 1960–2000.

The number of communities identified in each network is presented in Table 4. There is a noticeable fluctuation in the number of communities in the top1 networks. For example, in Top1D, a disordered fluctuation concerning the number of communities is notable, starting with 30 in 1960 and ending with 20 in 2000; this implies that globalization makes the architecture of Top1D less fragmented with modules that are more interconnected between them. On the contrary, we observe that in the Top1O the amount of communities increase from 15 in 1960 to 23 in 2000, which are driven by world events as well as increasingly selective immigration laws in developed countries and tend to generate significantly diversified migrant stocks. Moreover, when it comes to compare the number of communities with the number of components, we notice that considering the weight of the links reveals more about the inside of the network and the way the countries form communities. In both top1 networks, the number of components is less than the number of communities, showing again the importance of weight in characterizing the network. For the Top1D networks, the number of components show a downward trend (from 14 to 10) compared with the Top1O networks, which grows from 13 to 19 components.

**Table 4:** The number of communities and components in the international migration top1 networks.

		1960	1970	1980	1990	2000
Top1D	No. Communities	30	18	11	14	20
	No. Components	14	14	9	9	10
Top1O	No. Communities	15	17	16	20	23
	No. Components	13	17	16	20	19

### Community evolution

In the last part, we characterize the time-evolution of in-degrees for Top1D and out-degrees for Top1O, highlighting the way the nodes interact with each other during the five networks.

Table 5 shows the size of identified communities in terms of the number of nodes. There is an increased level of concentration in terms of the number of countries associated with a reduced number of communities. For example, in the 1960 decade, the top ten communities of the Top1D network includes 133 countries (that is, 59% from the total number of countries) in which the first 5 communities are made up of 91 countries. In the meantime, in the Top1O network, the first ten covers an impressive number of 212 countries (94% from the total number of countries) where the first five have 160 countries.

**Table 5:** The evolution of community size in the international migration top1 networks.

	Top1D					Top1O				
Community	1960	1970	1980	1990	2000	1960	1970	1980	1990	2000
1	43	67	88	81	76	40	34	45	43	31
2	18	35	34	29	21	39	30	36	33	24
3	11	18	32	15	16	31	29	33	26	23
4	10	13	18	15	14	25	28	22	24	23
5	9	12	14	14	12	25	23	22	16	15
6	9	12	12	14	11	22	20	14	13	11
7	9	10	10	13	10	16	17	12	9	10
8	8	10	8	10	8	6	11	10	9	10
9	8	9	5	9	8	4	8	6	9	10
10	8	9	3	8	6	4	6	6	7	9
11	8	6	2	6	6	4	4	5	7	8
12	7	6	---	5	6	4	4	4	6	7
13	6	5	---	4	6	4	4	4	5	6
14	6	4	---	3	6	1	3	3	4	6
15	6	4	---	---	5	1	3	3	4	6
16	6	2	---	---	4	---	1	1	3	5
17	5	2	---	---	3	---	1	---	3	5
18	5	2	---	---	3	---	---	---	2	4
19	5	---	---	---	3	---	---	---	2	4
20	5	---	---	---	2	---	---	---	1	3
21	4	---	---	---	---	---	---	---	---	2
22	4	---	---	---	---	---	---	---	---	2
23	4	---	---	---	---	---	---	---	---	2
24	4	---	---	---	---	---	---	---	---	---
25	4	---	---	---	---	---	---	---	---	---
26	3	---	---	---	---	---	---	---	---	---
27	3	---	---	---	---	---	---	---	---	---
28	3	---	---	---	---	---	---	---	---	---
29	3	---	---	---	---	---	---	---	---	---
30	2	---	---	---	---	---	---	---	---	---

Furthermore, at the longitudinal level, this situation emphasizes again the role played by international migration in the first phase of the globalization phenomenon, involving an opposite trend regarding the concentration of communities in the two top1 networks. In order to support this argument, we look at the level of concentration in the first 5 communities of each top1 network. With an increasing trend (i.e., evolving from 91 countries in 1960 to 139 countries in 2000), the Top1D network shows the highest concentration of node in 1980 reaching 186 countries (that is, 82% from the total number of countries). On the contrary, the Top1O network shows a negative trend, evolving from the pick of 160 countries in 1960 (that is, 70.8% from the total number of countries) to 116 countries in 2000. Both networks start in 1960 with around 40 nodes and evolve to 76 in the Top1D network compared with the Top1O network, which evolves to 31 nodes in 2000.

We conclude that in terms of the evolution and structure of communities, the situation is complex. While top1 networks include communities that grow from decade to decade and are able to absorb or to dissolve small communities, they also include more stable communities during that time. Furthermore, once the communities structure is determined, we can investigate the node’s degree evolution as the potential cause of the progressive disappearance for the legacy of old communities within the succeeding decades.

In Table 6, we summarize the statistics of degrees in the case of both top1 networks. Needless to say, more connected nodes tend to be more central. We notice that only a few countries (an average of 4.86% for Top1D and 3.54% for Top1O) have a high (that is, 5 or more) number of degrees. These so-called central countries are in a different number for the two top1 networks. For example, in the Top1D network, this number fluctuates between 13 and 9 compared with the Top1O network, which fluctuates between 9 and 6. Both top1 networks registered a peak in 1970 with 14 and 10 central countries, respectively. Furthermore, it is interesting that more than half of the total number of countries (around 70% for Top1D and 56% for Top1O) do not share any degrees over time (i.e., Degree = 0). Also, in this case, the evolution over time is different in the two top1 networks. For example, with a positive evolution from 147 to 158 compared with the negative trend from 134 to 122 countries, the Top1D network shows a higher concentration compared to the Top1O network.

**Table 6:** Degree statistics in international migration top1 networks.

	1960		1970		1980		1990		2000	
Degree	NC	%	NC	%	NC	%	NC	%	NC	%
**“in-degree” in Top1D**										
> = 5	13	5.75	14	6.19	10	4.42	9	3.98	9	3.98
4	2	0.88	6	2.65	3	1.33	6	2.65	4	1.77
3	11	4.87	2	0.88	8	3.54	7	3.1	6	2.65
2	10	4.42	15	6.64	11	4.87	11	4.87	13	5.75
1	43	19.03	40	17.7	31	13.72	27	11.95	36	15.93
0	147	65.04	149	65.93	163	72.12	166	73.45	158	69.91
**“out-degree” in Top1O**										
> = 5	9	3.98	10	4.42	8	3.54	7	3.1	6	2.65
4	2	0.88	1	0.44	2	0.88	2	0.88	3	1.33
3	13	5.75	9	3.98	10	4.42	13	5.75	16	7.08
2	15	6.64	19	8.41	24	10.62	26	11.5	22	9.73
1	53	23.45	59	26.11	52	23.01	54	23.89	57	25.22
0	134	59.29	128	56.64	130	57.52	124	54.87	122	53.98

Notes: NC: Number of countries sorted according to the number of degrees (for the Top1D network, they we sorted after the in-degrees; for the Top1O network, they were sorted after the out-degrees); %: Proportion is the result of number of countries (NC) in the total number of countries considered in the analysis (226).

In the final analysis, we identify the 25 most central countries that present the highest number of in- and out-degrees in each top1 network at every decade between 1960 and 2000 (see Table 7). It is interesting to note that in these 25 most central countries, 9 countries are developed and 16 are developing, and they exhibit different roles in each top1 network. Specifically, the Top1D network has more developed countries and shows an increased trend over time, while Top1O includes some large developing countries with high populations, like China and India. In the top1 destination networks, all these countries have not less than one in-degree in at least 3 of 5 years. The in-degree shows how many countries have these countries as their topmost destination according to migrant stock. In top1 origin networks, all the countries have more than 1 degree in all of the 5 years. The out-degrees present how many countries have this country as their topmost origin after migrant stock. As we can see, the countries with higher in-degrees in the Top1D network show an evolution that is not very stable during the time compared with the out-degrees in the Top1O network.

**Table 7:** The in- and out-degrees for major countries in the international migration top1 networks.

“in-degree” in Top1D						“out-degree” in Top1O					
Country	1960	1970	1980	1990	2000	Country	1960	1970	1980	1990	2000
USA	37	32	58	58	60	GBR	16	18	18	14	13
FRA	12	7	21	14	11	RUS	13	14	14	15	15
RUS	4	8	10	13	13	FRA	12	12	16	13	11
DEU	3	9	10	8	13	IND	11	11	10	10	11
GBR	8	10	9	10	6	CHN	11	8	10	9	8
AUS	5	8	6	9	8	USA	10	10	8	6	7
ZAF	8	8	5	7	7	DEU	9	7	6	3	2
IND	5	4	6	8	7	ITA	6	6	3	3	2
NZL	6	9	4	4	5	RWA	4	5	3	5	2
PSE	9	6	5	4	4	ESP	6	5	3	3	2
ARG	8	5	4	4	4	HTI	3	3	4	4	4
TTO	9	9	2	0	0	ZAF	3	3	3	4	3
CIV	3	5	3	5	3	COL	2	3	3	3	4
CAN	3	4	2	4	3	GIN	3	3	3	3	3
UGA	5	5	3	1	1	PRT	2	3	4	3	3
ETH	3	3	2	3	3	SEN	3	3	3	3	3
NCL	3	4	2	3	2	UKR	3	3	3	3	3
CMR	3	4	3	2	1	IDN	3	2	2	3	3
NGA	0	0	7	2	4	MAR	2	4	2	2	3
ZWE	3	4	2	2	2	AUS	3	1	2	3	3
ISR	5	4	3	0	0	PSE	1	1	5	2	3
PRT	0	2	3	4	3	DNK	2	2	2	3	2
KWT	0	3	3	4	1	HND	3	2	2	2	2
PAK	1	5	2	1	1	AGO	2	2	2	2	2
SAU	0	0	3	3	4	CIV	2	2	2	2	2

Notes: Countries are sorted according to the total number of degrees in the 5 networks; in-degrees in the Top1D network indicate how many countries have this country as their topmost significant destination for emigrants; out-degrees in the Top1O network indicate how many countries have this country as their topmost significant origin for immigrants.

Next, we will focus our attention on the 5 topmost central countries in top1 networks. The top tier includes 4 of the largest countries with the highest population such as USA, Russian Federation, China, and India but also the West European countries including France, Germany, and the United Kingdom. More precisely, the countries addressed are the most central countries in the first top5 communities in both top1 networks. In order to understand the position played by the most central country in the communities, we will take the example of USA. In 1960, USA was bringing to the community 37 nodes from the total of 43. In 1980, the size of the community grew to 88, in which USA brought 56 nodes, even though it was considered the main destination for 58 countries. The remaining two countries (i.e. Germany and France) formed their own communities.

The connection with their former colonies was essential for Britain and France; at every decade between 1960 and 2000 this was characterized by a fluctuated evolution in Top1D networks and a relatively stable one in Top1O networks with a larger number of countries of destination for British emigrants. The topmost number of British emigrants went to Canada in 1960, followed by Australia for the remaining years. In the meantime the French emigrants considered Morocco in 1960, Democratic Republic of the Congo in 1970, and USA in 1980, 1990, and 2000. In top1 destination networks, the second most central country is France, with the highest overall in-degree coming from Algeria compared with the United Kingdom, who received the highest number of immigrants from India in 1960, and Ireland in the rest of the decades under consideration. In the meantime Germany played an important role mostly in top1 destination network receiving the highest number of immigrants from Poland in the first four decades and Turkey in 2000.

On the other hand, compared with the Western European countries, the large-scale migration to the USA developed later due to restrictive legislation enacted in the 1920’s [41]. USA’s immigrants in 1960 came from 37 countries, and they grew steadily after the 1970 and reached 60 countries by 2000. During that time, the highest number of immigrants were from Italy in the 1960s and 1970s, followed by Mexico in the 1980s, 1990s, and 2000s.

Asian migration is not new. Even so, the discriminatory rules of the countries that repealed against Asians [41] has made from-migration within Asia a more popular trend being clearly captured in the Top1O network. In the nineteenth century, China’s main destination country in 1960 and 1970 was Indonesia followed by Hong Kong in the remaining years. China’s out-degree decreased from 11 countries in 1960 to 8 in 2000, and all of the destination countries during the time were countries inside Asia. India, which presented a stable evolution during that time, was considered the topmost origin country for 11 nodes (with the exception in 1980 and 1990 only 10 nodes). With the highest number of emigrants going to Pakistan in all the 5 years, India displayed the shortest path to countries located in southern Asia (i.e., Bhutan, Nepal, Sri Lanka, Bangladesh) but also in Persian Gulf (i.e., Oman, United Arab Emirates, Saudi Arabia).

The collapse of the Berlin Wall in 1989 followed by the collapse of the Soviet Union in 1991 and the Eastern European socialist states led to instability in Central Europe and created a threat to Western Europe in terms of migration [41]. Millions of people have moved in and between the successor states of the former Soviet Union, making immigrants from the Russian Federation the central country of origin for 13 countries in 1960 and 15 in 2000. It is interesting to note that over the five decades, the largest number of immigrants in the Russian Federation were from Ukraine, and the largest number of Russian emigrants had Ukraine as their destination country.

All this helps to infer that the Top1D network changed more than the Top1O network due to world events and increasingly selective immigration policies in developed countries that led to a higher diversification of migration stocks.

## Conclusions

This paper detects the communities structure in international migration top1 networks from the perspective of destination and origin countries, respectively. This study is different from previous studies that have focused largely on the level of country-to-country flows and on the global migration network directly; this new approach provides the possibility to study the migration network from a more concise and clear perspective to demonstrate communities dynamics and how the migration behavior of countries changes over time.

Our exercise showed that through building top1 networks, we were able to focus on the relationships between all pairs of countries while covering about 50% of the complete migrant network stock. One of the key observations of our study is that many links in international migration top1 networks are stable. This implies that even though the stability is around 80%, the difference of 20% is significant enough to change the architecture of the communities. The results showed that both top1 networks had clear but different community structures with opposite trends. Therefore, the Top1D network shows a downward trend in the number of communities exhibiting shorter paths, mainly to developed countries, compared with the Top1O network, where the communities are not so highly concentrated and present longer paths and more stable groups around both developed as well as more substantial developing nations.

Also, our analysis revealed that only few countries have a central role in the communities evolution, with patterns becoming more skewed to migration from an increasing diverse array of origin countries concentrating on a shrinking pool of destination countries, which are mostly developed (e.g., USA and United Kingdom) but also developing countries such as the Russian Federation and India. Dissolution of the USSR in fifteen ethnically based national republics has led to many Russians who suddenly have become minorities in the new state to migrate to Russia. The communities structure also reveal the relation between the United Kingdom and France with their former colonies all over the world. Concerning the origin of the immigrants, both developed and developing countries were shown to participate in the migration process, with a decreasing evolution during the period of study for Western European countries and USA and a stable evolution for the countries with large populations, such as Russia, India, and China.

This analysis can be extended in several ways by finding explanations to the following questions: Why are top1 selection actions stable for some countries, and why do some change? What causes stability? Moreover, we can build a model to investigate the factors that influence the trend in top1 networks. Future research should extend the extraction methodology for determining how the migrants change their preferences in choosing the country of destination by considering not only the highest link weight (e.g., in top1) but the first two or three (e.g., generating top2 and top3 networks). Furthermore, we can explore the communities to determine the economic and social effects. In addition, the methodology presented in this paper can also be applied on other directed weighted networks, such as the international trade network and the international investment network.
